# Natural nanofibers embedded in the seed mucilage envelope: composite hydrogels with specific adhesive and frictional properties

**DOI:** 10.3762/bjnano.15.126

**Published:** 2024-12-13

**Authors:** Agnieszka Kreitschitz, Stanislav N Gorb

**Affiliations:** 1 University of Wrocław, Department of Plant Developmental Biology, ul. Kanonia 6/8, 50-328 Wrocław, Poland; 2 Kiel University, Department of Functional Morphology and Biomechanics, Am Botanischen Garten 9, D-24098 Kiel, Germany

**Keywords:** adhesion, cellulose, friction, hydrogel, mucilage envelope, seeds

## Abstract

The increasing interests in natural, biodegradable, non-toxic materials that can find application in diverse industry branches, for example, food, pharmacy, medicine, or materials engineering, has steered the attention of many scientists to plants, which are a known source of natural hydrogels. Natural hydrogels share some features with synthetic hydrogels, but are more easy to obtain and recycle. One of the main sources of such hydrogels are mucilaginous seeds and fruits, which produce after hydration a gel-like, transparent capsule, the so-called mucilage envelope. Mucilage serves several important biological functions, such as supporting seed germination, protecting seeds against pathogens and predators, and allowing the seed to attach to diverse surfaces (e.g., soil or animals). The attachment properties of mucilage are thus responsible for seed dispersal. Mucilage represents a hydrophilic, three-dimensional network of polysaccharides (cellulose, pectins, and hemicelluloses) and is able to absorb large amounts of water. Depending on the water content, mucilage can behave as an efficient lubricant or as strong glue. The current work attempts to summarise the achievements in the research on the mucilage envelope, primarily in the context of its structure and physical properties, as well as biological functions associated with these properties.

## Introduction

The definition of hydrogels describes them as hydrophilic, three-dimensional (3D), polymeric networks able to absorb huge amounts of water [1–3]. This term refers perfectly to the mucilage envelope produced by many fruits and seeds (diaspores) of diverse plant taxa [4–9]. Mucilage is considered as a natural hydrogel and shares specific features with synthetic hydrogels [2,9–11]. Hydrogels are 3D networks of polymers (i.e., polysaccharides in plant seeds) interacting via chemical bonds (ionic and covalent), physical interactions (hydrogen bonds), or van der Waals forces [3,11–12].

**Figure 1:**
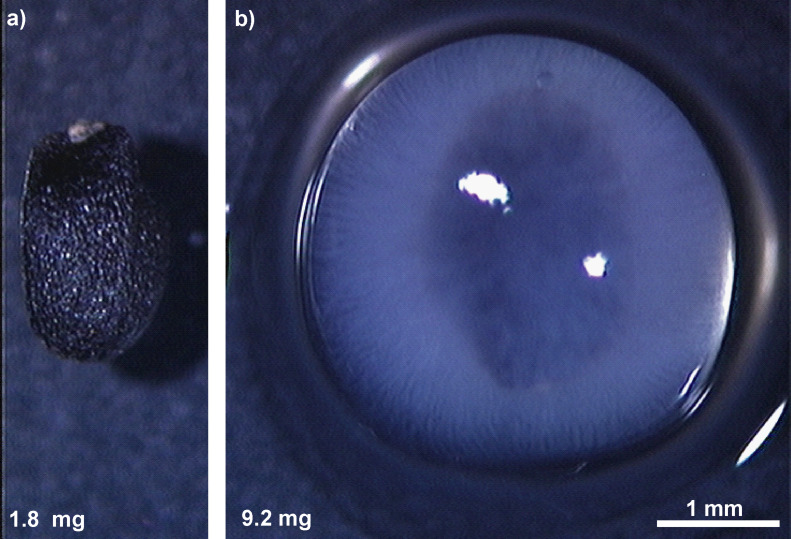
*Ocimum basilicum*. (a) Dry seed and (b) seed after hydration with visible mucilage envelope. The mucilage envelope accumulates water and keeps it around the seed. Note the mass increase after hydration.

The ability to produce the mucilage envelope is a widespread feature in diverse plant groups (mosses, ferns, gymnosperms, and dicotyledons) as well as plant organs (roots, leaves, flowers, seeds, and fruits) [4–6]. Among the various substances produced by diaspores, mucilage at the macroscale can be very easy to perceive without any special equipment. Macroscopically observable mucilage is often a transparent, gel-like capsule formed around the diaspore after hydration with water (Figure 1).

At the microscale mucilage exhibits, before hydration, successive layers formed by adcrustation in the mucilaginous cells of the seed/fruit coat (the outermost covering of the diaspore) [6,13]. Mucilage is often composed of three types of polysaccharides, which are typical components found in the plant cell wall. It exhibits modified secondary cell wall, where pectins are the dominating component, while hemicelluloses and cellulose fibrils constitute the lesser part of its composition [14–16]. The nanoscale level of the spatial organisation of mucilage observed with scanning electron microscopy (SEM) reveals the complexity of the mucilage with special features, such as 3D organisation of polysaccharides in a net-like structure [7,13].

In the last years, the mucilage produced by plant diaspores became of high interest in diverse sectors, such as medicine, cosmetics, food, biomedicine, pharmaceutics, nanomaterials, and bioinspired nanotechnology [11,17–20]. Mucilage is a natural, biodegradable, non-toxic plant product, odourless, colourless, and tasteless [11,21]. Its chemical composition and special physical properties allow many applications of mucilage, for example, as thickening and structuring (gel-forming) agent, emulsifier or stabiliser for food products, scaffold for tissue regeneration, additive in formation of medicinal tablets, and for gels and wound dressings [15,18,20–22].

Mucilage is a complex mixture of polysaccharides, that is, pectins, hemicelluloses, and cellulose fibrils, which are derivatives of the modified secondary cell wall with its special properties. Among these properties, one of the most important ones is the ability to accumulate a large amount of water [2,6,16,23]. This is connected to many ecological advantages for the diaspores. The mucilage supports seed germination, dispersal, and various interactions with other organisms. It protects diaspores against digestion, pathogens, harvesting by ants, and osmotic stress [4–6,10,24–25].

Depending on the hydration level, mucilage exhibits distinct physical properties, which are also connected with its biological functions. In a fully hydrated state, it demonstrates very low friction, important for example, in endozoochoric diaspore dispersal [26–28]. Hydrated mucilage can very strongly adhere to surfaces (e.g., stone or glass) when completely dried out after contact, with pull-off forces reaching values around 6.5 N [29]. Such strong adhesion can enable seed attachment to the soil, preventing removal and damage by other organisms [16,30], or to animal bodies, promoting epizoochory [31–33]. These distinct physical features make mucilage also an important substrate for pharmaceutical, biomedical, and food industries [11,15,19–21].

**Figure 2:**
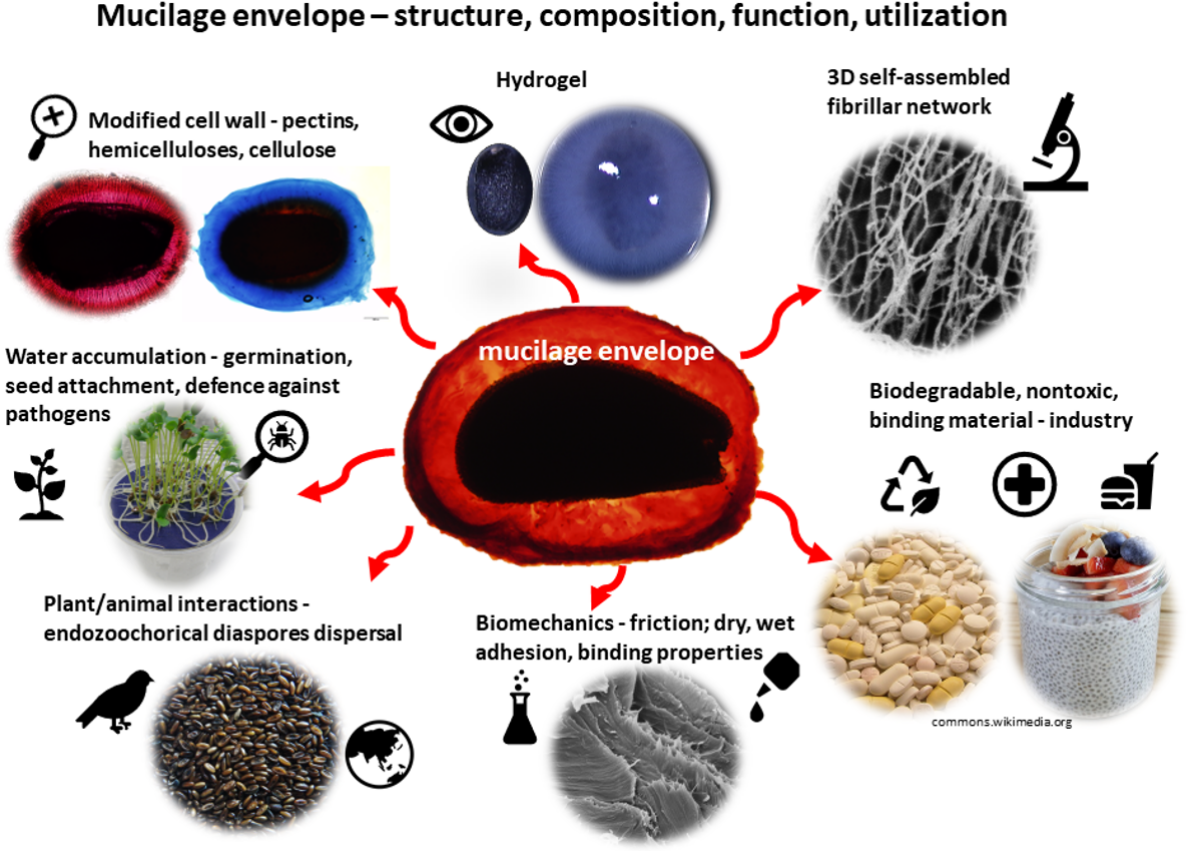
Structure, composition, function, properties, and exploitation of the seed mucilage envelope. The photo of Chia pudding with coconut milk and berries was adapted from https://www.flickr.com/photos/197996902%40N06/52774529156/, (“Chia pudding with coconut milk and berries (KETO, LCHF, Low Carb, Gluten free, FIT)“, © Ewelina Podrez-Siama, published via Flickr, distributed under the terms of the Creative Commons Attribution 2.0 Generic License, https://creativecommons.org/licenses/by/2.0). The photo depicting pills was adapted from https://commons.wikimedia.org/wiki/File:Tablets_pills_medicine_medical_waste.jpg (“Various unused tablets collected to be treated as medical waste“, © Pöllö, published via Wikimedia Commons, distributed under the terms of the Creative Commons Attribution 3.0 Generic License, https://creativecommons.org/licenses/by/3.0/).

Here, we briefly review the basic composition and structure of mucilage, its frictional and adhesive properties, and ecological aspects associated with these properties. We also summarise and discuss the results of our studies from the last few years conducted on mucilage envelopes and summarise them to demonstrate the current state of knowledge on this topic (Figure 2).

## Review

### Spatial structure of the mucilage – from pressed layers to a 3D network

Substances with gel character (slime, mucus, and mucilage) are ubiquitous in nature and are produced by diverse organisms such as bacteria, plants representing diverse groups (algae, ferns, and higher plants), and animals (fishes, frogs, and jellyfish). They can be important in different ways for the organisms (locomotion, reproduction, and defence) [34–35], but one of their most significant functions is their ability to absorb water [6,11,15,17–18]. One of such gel-like natural materials studied intensively over the last years is the diaspore (seeds and fruits) mucilage envelope produced by different plants (monocotyledons and dicotyledons) [2,4,6,16,22,36–37]. The mucilage envelope can be described as nanoscale 3D self-assembled fibrillar network, which is able to entrap water and to form a so-called molecular gel after hydration [8,22,38].

**Figure 3:**
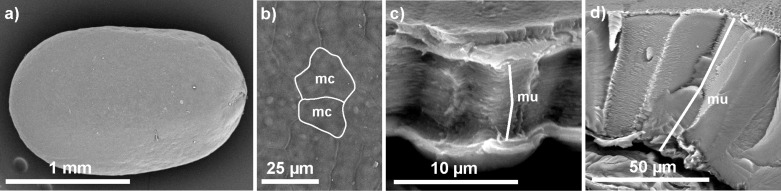
*Salvia hispanica* dry seed. (a) The whole seed is covered with mucilaginous cells. (b) The mucilaginous cells (mc) can have irregular shape (white outlines). (c, d) Cross fractures through the mucilaginous cells; a very thick mucilage deposit (mu) composed of many thin layers is visible in the dry stage of the mucilaginous cell wall.

**Figure 4:**
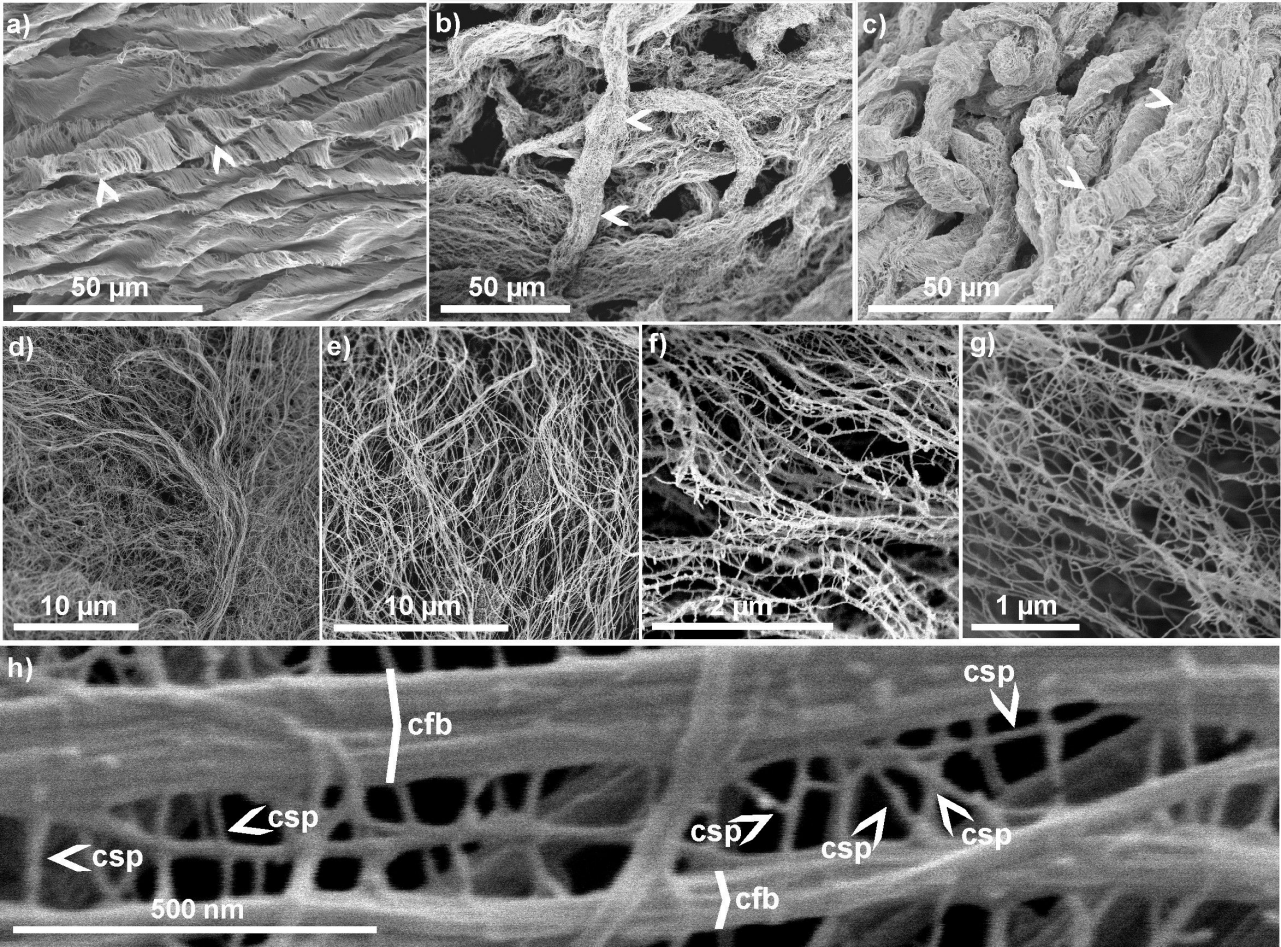
Mucilage envelope after hydration and critical point drying, visualised in SEM. (a) *Ocimum basilicum*. (b) *Salvia sclarea*. (c) *Commicarpus helenae*. (d) *Artemisia annua*. (e) *Ocimum basilicum*. (f) *Salvia sclarea*. (g) *Plantago psyllium*. (h) *Ocimum basilicum*. (a–c) Mucilaginous cell walls after hydration and subsequent mucilage expansion can present a tubular form. (d–g) After complete expansion of the envelope, the mucilaginous polysaccharides (pectins, hemicelluloses, and cellulose fibrils) form a netlike 3D structure. (h) High-magnification image showing cellulose fibril bundles (cfb), which form the main (unbranched) skeleton, while pectins and hemicelluloses (csp – cross-linking polysaccharides) are the cross-linkers. This structure is typical of diverse taxa from different plant groups and represents a characteristic 3D netlike architecture of a hydrogel.

Mucilage, which is produced by the mucilaginous cells of diaspores in a form of densely packed layers, has the ability of loosening its structure after hydration into an easily accessible 3D fibrillar network [7,13–14,39] (Figure 3 and Figure 4 below). One of the key components of this network are, in general, cellulose fibrils, which constitute a kind of skeleton for other polysaccharides (pectins and hemicelluloses); however, there is also almost purely pectic seed mucilages without cellulose fibrils and mucilage dominated by hemicelluloses [6–7]. This self-organising nature and the natural composition makes the mucilage envelope a perfect material for diverse studies and a model for the production of synthetic gels or gel-like substances with properties resembling those of hydrogels [11,22].

Cellulose nanofibrils (CNFs) have been of interest for diverse industry branches because of their natural origin and biodegradability. They are used, for example, for paper production, as food additives, in biofilms, and in the production of packing materials and aerogels [15,18,20–22]. However, little is known about the structural properties of CNFs, and they require proper characterisation of their micro- and nanostructures. In the last years, SEM visualisation, combined with the critical point drying (CPD) procedure, has been widely used in nanostructural studies of diverse hydrogel-like samples, containing cellulose fibrils, or biofilms [7,40–41]. The CPD method allows one to maintain the original 3D ultrastructure of the samples without collapsing after dehydration [40–41]. When hydrated hydrogel-like CNFs are gently dried using CPD, the result is an aerogel-like material [41–42] with extremely interesting frictional properties [43].

The cell wall is a typical part of plant cells, and its basic chemical composition includes cellulose, hemicelluloses, and pectic polysaccharides [44–47]. Cellulose is a linear polymer composed of β-1,4-linked ᴅ-glucose [47]. The cellulose chains are held together by intramolecular hydrogen bonds, forming cellulose microfibrils, whose diameter can vary across species from 2.2–3.6 nm [48–51], over 3–4 nm [52], to even 30 nm of average width [53]. Microfibrils are typical of primary cell walls, while the next higher level of organisation are macrofibrils [54], specific for secondary cell walls [47,55–56].

Cell wall architecture has been studied in many cases on the primary cell wall of parenchymatous, root tip, or epidermal cells [54,57–59]. Visualisation of the secondary cell wall was previously carried out for tracheary elements of xylem [60–62]. The microfibrils of the primary cell wall are rather thin (2–3 nm), which makes their differentiation from other polysaccharides (pectins and hemicelluloses) rather difficult. The size of the microfibrils of the secondary cell wall (20–30 nm) [53] makes their observation easier, particularly using high-resolution microscopy techniques, such as atomic force microscopy (AFM), transmission electron microscopy (TEM), SEM, or cryo-SEM [45,57,63–66]. Very often, the procedures for preparing mucilage envelope samples can destroy and/or influence the organisation of polysaccharides, making the analysis of spatial structure of the mucilage impossible. The complicated preparation procedures and analysis give us often an information limited to just one factor, for example, to specific chemical composition or topology (AFM, FTIR, or Raman microscopy) [45]. Ideally, the comparison of data from diverse visualisation techniques can provide us with reliable results about the 3D organisation of the polysaccharides within the mucilage envelope.

CPD is a technique used for diverse biological samples (plants, animals, and microorganisms) that are very fragile and contain water. CPD allows for drying of samples without deforming them or collapsing the structure. This technique is very effective for sample imagining in TEM and SEM [7,41,67–68]. CPD minimises the negative pressure differences during drying. The comparison of CPD and air-drying techniques of plant material, for example, parenchymatic cells [69] and the mucilage envelope [7,13], clearly showed advantages of the CPD method. During air-drying, the mucilage envelope changes from its initial form (a soft hydrated gel) into a thin, compressed, crust-like layer [7]. Similarly, parenchymatic cells were completely flattened [69] after air-drying. CPD-dried mucilage envelope preserves its 3D structure and form [7,13]. Another example of the advantages of CPD are studies of TEMPO-oxidised cellulose nanofibrils (TCNFs). In these structural studies, freeze-drying and critical point drying techniques were applied to preserve the open fibril structure of gel-like TCNFs. The result of gently drying the hydrated CNFs was an aerogel-like material with large surface area [41]. The freeze-drying process causes the formation of ice crystals, which destroy the delicate ultrastructure of the studied material [70].

As mentioned before, the mucilage envelope represents a special type of secondary cell wall, where non-cellulosic polysaccharides dominate (pectins and hemicelluloses) and cellulose is a minor, but nevertheless important, skeletal component [2,6–7,71]. The cellulose fibrils appear as an important structural element in the seed mucilage envelope for plants from diverse genera, such as *Artemisia annua*, *A. ballerieri*, *A. campestris* (Asteraceae), *Arabidopsis thaliana*, *Capsella bursa-pastoris*, *Lepidium sativum* (Brassicaceae), *Ocimum basilicum*, *Salvia hispanica* (chia), *Salvia sclarea* (Lamiaceae), or even in such an exotic genus like *Commicarpus* (Nyctaginaceae) [4–7,72–73]. The observation of the mucilage envelope is easily possible at the macroscale with the naked eye. The hydration of the seed causes the formation of a transparent gel-like envelope surrounding the diaspore [7,73–74] (Figure 1), which is easily observable. Air-drying of hydrated mucilage causes water evaporation and its compression to a transparent, thin layer tightly adhering to substrates (stone or glass). In this form of mucilage, long, tangled or parallel organised cellulose fibrils can be recognised under the high magnification of a SEM [7,13,73]. The observation of the 3D nanoscale organisation of the mucilage envelope requires critical point drying, as discussed above (Figure 4). From CPD+SEM visualisations, the probable localisation of mucilage polysaccharides has been deduced based on their shape, thickness, and position in the complex netlike structure [7]. The revealed architecture of the mucilage envelope [7] resembles the known structure of cell walls [57,74–78]. The long, unbranched fibrils (main chains) are cellulose fibrils building the main skeleton of examined mucilage. Their average size ranges from 20.8 nm in *Arabidopsis thaliana*, over 32.7 nm in *Salvia hispanica*, to 57.3 nm in *Ocimum basilicum* [7]. TEM and SEM [45,65,78–79] showed the size of cellulose microfibrils in a range of 3–50 nm, depending on cell wall type. This wide range of size can be also a result of bundles formed by cellulose fibrils (Figure 4h) [80]. The results of our research [7,13] confirmed the diversity of cellulose fibril sizes in the mucilage envelope of different taxa and also the presence of cellulose bundles (Figure 4).

Another interesting and effective method to visualise mucilage hydrogels in three dimensions at the nanoscale is freezing-drying (lyophilisation). Samateh et al. [8] used the mucilaginous seeds of *Ocimum basilicum* and *Salvia hispanica* to study the 3D organisation of mucilage (called there *molecular gel*). The lyophilisation allowed for carefully removing the water from the mucilage while preserving the polysaccharide fibrils in the extended state. SEM imagining of *S. hispanica* mucilage revealed the network of fibrils extending from the seed surface into all directions. The diameters of these fibrils were estimated to be around 50 nm (in SEM) and around 20 nm (in TEM) [8]. Our results for the related species *Salvia sclarea* showed a mean values of 32.7 nm (range 24.7–44.2 nm) for the main chains (cellulose fibrils) and 18.4 nm (range 14.1–23.8 nm) for the cross-links (pectin and hemicellulose chains) [7]. One difference can be seen when comparing both methods. In our studies, we observed small granules covering the fibrils. We supposed that they are proteins that are natural elements of the cell wall and the mucilage envelope [7,13,16]. Samateh et al. [8] did not detect them. This can be a result of the prior treatment, such as dehydration in alcohol series before CPD (possibly an effect of the protein denaturation), or due to differences in chemical composition between the taxa studied.

Critical point drying in studies of natural samples containing cellulose fibrils [40–41,81], other polysaccharides (cell wall, mucilage, and envelope) [7,13], and synthetic hydrogels [82] maintained the 3D network of the studied materials. During drying, the hydrogen bonds undergo reformation, which may cause the mechanical collapse of the spatial structure.

### Frictional properties

One of the important features of hydrogels [83–87] is their ability to decrease friction in contact. The frictional properties of hydrogels depend on their chemical composition [26–27,88–89], on monomer and cross-linking concentrations, and on the type of substrate surface [88].

**Figure 5:**
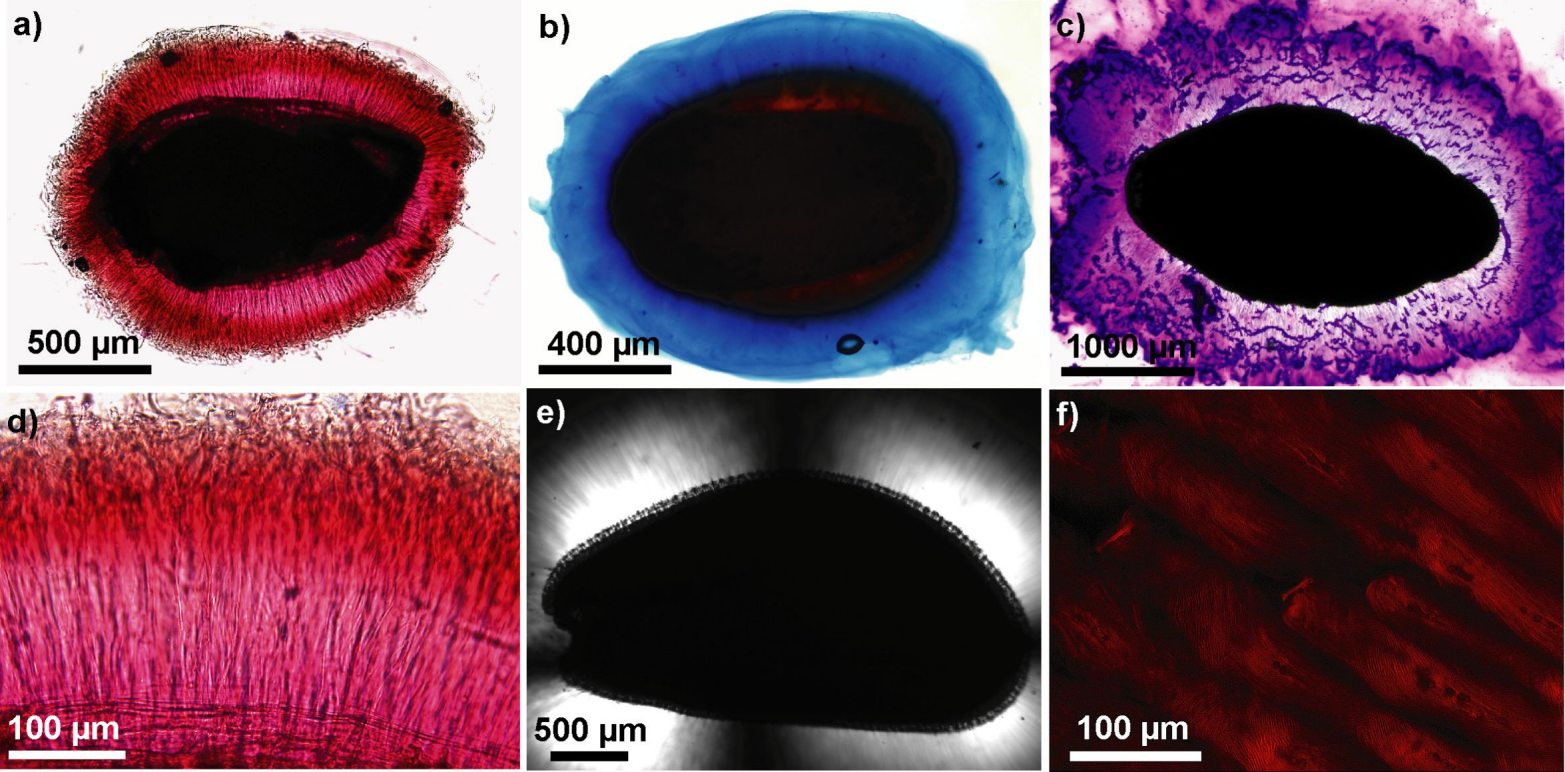
Staining of basic mucilage components. (a) *Artemisia annua* – pectins stained with ruthenium red. Delicate cellulose fibrils are visible stretching radially from the seed surface. (b) *Capsella bursa-pastoris* – pectins stained with alcian blue. (c) *Plantago ovata* mucilage is rich in hemicelluloses but comprises also pectins – staining with crystal violet. (d) *Artemisia annua* – magnification of the mucilage envelope. Delicate cellulose fibrils, stretching from the seed surface, are imbedded in the mass of pectins. (e) *Lepidium sativum* – polarisation microscopy image of cellulose fibrils demonstrating the presence of crystalline cellulose shining white. (f) *Ocimum basilicum* – fluorescence microscopy image of the mucilage stained with direct red. Cellulose fibrils are stained red (compare with Figure 5a).

Hydrogels with their low friction are crucial in biomedical applications or for drug delivery [38,83,86,88]. The diaspore mucilage is regarded as a natural hydrogel [38] because of its capacity to absorb water and to form specific netlike spatial architecture of interlaced polysaccharides (see above). The most important polysaccharides of the mucilage with hydrophilic characters are pectins and hemicelluloses. The former are composed of negatively charged galacturonic acid residues responsible for the high hydration ability of pectins [90] and are the main component of mucilage in diverse taxa of such genera as *Artemisia*, *Arabidopsis, Lepidium,* and *Linum* (Figure 5a–c) [5–6,90]. Among hemicelluloses, heteroxylans are extremely hydrophilic components, which dominate the seed mucilage in *Plantago* taxa [2,91]. Another important component of the mucilage is cellulose (Figure 5a,d–f). Its ability to bind water is not as great as that of pectins, but it plays an important structural role and can interact with other polysaccharides in mucilage, forming the 3D network [7]. The ability of a hydrogel or mucilage to absorb water is due to the presence of hydrophilic groups, such as carboxyl or hydroxyl groups. At the same time, hydrogels are insoluble in water because of their cross-linked network structure [38,92]. Two main physical properties of mucilage, friction and adhesion, are directly connected with the amount of water present in the hydrogel. Depending on the hydration degree of mucilage, it can behave as super lubricant [93–94] or super glue [29].

### Friction of the hydrated mucilage envelope

Many plant hydrogels possess very good lubricating properties, sometimes better than those of artificially produced substances [94]. Some of them reveal even super low friction, that is, frictional coefficients (µ) below 0.01 [95]. Exemplary values are μ < 0.03 in the seed mucilage of quince fruits [96], µ = 0.003 in polysaccharides extracted from red microalgae [97], and µ = 0.005 in mucilage of the water plant *Brassenia schreberi* [94]. *Ocimum basilicum* seed mucilage also exhibits super lubricity with a friction coefficient of 0.003 [94,98].

Experimental tests with mucilaginous diaspores after seed hydration demonstrate how the frictional properties of these diaspores change before and after contact with water and subsequent mucilage formation. The friction coefficient of dry flax diaspores was reported to be in the range of 0.499–1.073 measured on diverse substrates [99]. In our experiments, we obtained values between 0.25 and 0.31 on a glass surface [26]. The situation changed visibly after hydration. The mucilage envelope is very slippery, and the lowest friction coefficient occurs in the fully hydrated state, just at the end of the mucilage envelope formation. The measured friction coefficient values ranged from 0.039 to 0.055 (*Linum usitatissimum* (flax) seed mucilage) [26]. Flax seeds produce pectic mucilage, which is rich in rhamnogalacturonan I (pectin) and arabinoxylan (hemicellulose) [100–101]. The mixture of these polysaccharides gives the flax mucilage viscous properties [101]. Another example is the cellulosic mucilage of *Plantago lanceolata*. Plantain cellulose mucilage contains pectins, hemicellulose, and cellulose fibrils [27,102]. However, the content of cellulose in this mucilage is relatively low [27]. The cellulose fibrils here play an important role in helping to attach the mucilage to the seed surface and in preventing it against the loss from the seed [7,27]. The friction coefficient of *P. lanceolata* dry seeds ranged from 0.4 to 0.5 on a glass surface; for hydrated seeds with freshly formed mucilage, it ranged from 0.05 to 0.08 [27].

Another example of ultralow friction coefficient on glass was measured for *Ocimum basilicum* mucilage with a mean value of 0.003 [94]. The mucilage composition of *O. basilicum* differs clearly from those of *L. usitatissimum* and *P. ovata*. In *O. basilicum,* it is dominated by two hemiceluloses, glucomannan (43%) and xylan (24.29%) [94,103], but also contains a few percent of glucan [94,103] and arabinogalactan [94]. The mucilage of basil seeds also contains pectins, cellulose fibrils, and starch grains [7]. The cellulose fibrils here constitute a scaffold for other polysaccharides [7]. Friction measurements were also carried out with dry basil seeds, as well as with hydrated and dried (vacuum freeze-drying) seeds. The friction coefficient of dry seeds was ten times larger (0.02) than that of hydrated seeds with mucilage (0.002). The ultralow friction coefficient of basil mucilage was explained by the specific mucilage composition, that is, the presence of abundant OH groups in the polysaccharide molecules, a 3D network microstructure forming cross-linking sheets, and the presence of water [94]. The authors observed that a thin layer of basil mucilage was adsorbed at the glass surface because of the abundant OH groups. This layer prevented the mucilage from the direct contact with the glass, which finally resulted in ultralow friction [94]. Water, which forms hydration layers, one adsorbed at the mucilage surface and the other one at the glass surface, plays important role in ultralow friction [94]. During sliding, the hydration layers act as lubricant reducing friction [88,94].

We suppose that an important factor regarding ultralow friction is the presence of cellulose fibrils as the structural element reinforcing the mucilage. During the friction measurements on flax and plantain seeds, we observed that the pectic flax mucilage adhered to the glass plate and was almost lost. This probably happened because of the lack of cellulose fibrils in flax as it rarely occurred in cellulosic plantain mucilage [26–27]. Cellulose fibrils constitute a kind of scaffold for other components of mucilage; because of the interactions among all polysaccharides in the envelope they can remain on the seed surface [7]. We observed the lowest friction of mucilage just after hydration. In time series of measurements, the friction increased. The water loss from the mucilage caused an increase of its stiffness and adhesive properties [26]. The decreasing hydration level and the increasing viscosity of flax mucilage play an important role in the friction increase. The viscoelastic properties of mucilage in diverse flax lines were studied and showed that the proportion between xylan (hemicellulose) and uronic acid residues (pectin) can influence the viscosity of mucilage. A higher proportion of xylan leads to an increase of mucilage viscosity [104]. This feature is presumably responsible for the stronger adherence of the mucilage to the seed surface [101] and for higher friction [26]. The friction coefficient of *Linum usitatissimum* (0.039) [26] was slightly higher than that of *Ocimum basilicum* (0.003) [94], and both values can be considered as ultralow friction.

Zhang et al. [94] assumed that with an increasing shear rate the viscosity decreases, and the polysaccharide organisation in the mucilage becomes more ordered. This presumably causes easy sliding of *Ocimum basilicum* mucilage. It can be summarised that different factors influence the frictional properties of mucilage, namely, chemical composition, viscosity, network structure, and water amount.

One of the important roles of the mucilage envelope is the protection of the diaspore against damage by digestive organs of animals [28]. It can be expected that mucilaginous seeds with ultralow friction better fulfil their biological role than mucilaginous seeds with just low friction, which was demonstrated in our experiment with pigeons (see below).

### Friction after chemical and thermal treatments

The antimicrobial activity of seed mucilage was previously described for *Linum usitatissimum* [105–106], *Salvia hispanica* [106–107], and *Lallemantia royleana* [108]. However, the mucilage extracted from diverse seeds, such as *Ocimum basilicum*, *Cydonia oblonga*, *Lepidium sativum* [109], and the abovementioned flax and chia, are also popular substrates for the production of biofilms or the encapsulation of medical substances. A very important advantage of diaspore mucilages are their antimicrobial properties [105–106,110–111].

Phenolic compounds are secondary plant metabolites, which demonstrate a wide range of structures, from simple molecules to polymeric compounds. They can be produced in diverse plant organs, such as seeds, fruits, flowers, and leaves, and are involved in the defence of the plant against herbivore animals, fungi, and viruses [112–113]. Phenolic compounds are very abundant substances in seeds, playing an important role in their development and maturation. They are accumulated in most cases in the seed coat [113] and can be released after hydration and accumulated in the mucilage envelope as in case of *Ocimum basilicum* [98] or *Lallemantia royleana* (balangu shirazi) seeds [108].

Phenolic molecules in the seed mucilage of *Ocimum basilicum* were detected in a very small amount, which raises questions about their role. The experiment answering this question can be an oxidation process of phenols, which happens at increased temperature (40 °C) [98]. One can suppose that the increase of the temperature within the mucilage envelope in the natural environment can happen spontaneously. The mucilage envelope can act as a lens focusing the light and locally increasing temperature [4]. The temperature increase possibly activates enzymes involved in the germination processes [5]. This could happen to *Ocimum basilicum* seeds (as well as to seeds of other plant taxa) under natural conditions. The direct consequence of the temperature increase is the oxidation of phenolic molecules and the accumulation at the air–water interface of the mucilage envelope. This process also has an influence on the frictional properties of basil seed mucilage. As our experiments showed, the oxidised mucilage demonstrated a higher friction coefficient than the fresh mucilage envelope. This result can be explained by stronger chemical interactions between the phenolic layer formed at the mucilage surface and the acrylic glass used for the friction measurements. In this way, the protective function of phenolic molecules, which are concentrated on the mucilage surface, can be maximised [98]. Thus, we assume that increased concentration of phenolic compounds at the mucilage interface has an important antimicrobial function for the seed. The mucilage envelope supports the germination through speeding it up, but also protects the seeds against pathogens probably also by the presence of these phenolic substances [72,98].

As we have mentioned above, natural polysaccharide-based hydrogels obtained from seed mucilage of flax, plantain, chia, basil, and quince found broad application in various fields, such as food, agriculture, and medicine [20,114–115]. Diverse other substances and systems with antimicrobial activity (antibiotics, essential oils, phenolics, and metal nanoparticles) can be incorporated into the mucilage hydrogels, which after some additional modifications can be used for the production of biofilms, encapsulation, or lubricants with medical application [106,110–111,116–117]. Our findings may also help to design pathogen-resistant lubricating biomaterials with low friction, which can be achieved in rather simply way by adding phenolic substances to the medical hydrogels [98].

### Adhesive properties

Seed dispersal is a crucial factor for many plants, allowing for the genetic continuity as well as for occupying new habitats. Through diverse mechanisms developed by the plants, seeds can be dispersed at a local scale (in the vicinity of mother plant) or transported over longer distances between islands or continents [28,118]. Plants developed diverse morphological mechanisms for the diaspore transport. One of the ways of promoting long-distance dispersal is the proper interaction between the plant diaspores and animals [118–120]. Epizoochory is the way of diaspore transport on animals due to the presence of interlocking structures or sticky viscous substances, which allow for attachment to the animal body [118–119,121].

The mucilage envelope is one of the factors enabling short- and long-distance dispersal of diaspores. Many authors working on mucilaginous seeds supposed that the mucilage envelope can support epizoochoric ways of seed dispersal [29,31–33]. However, direct experimental support of the mucilage role in epizoochory (e.g., epiornitochory) is only scarcely documented in the literature like, for example, the dispersion of fruits of *Adenostemma brasilianum* [121] or the human-mediated dispersal of *Plantago asiatica* seeds [122]. The mucilage envelope reveals its adhesive properties in a hydrated state. However, just after the seed hydration and mucilage envelope formation, the adhesion force is very low. With the loss of water, adhesion increases and determines the attachment potential of the diaspore to animal bodies [27–28]. Another factor regarding mucilage adhesion is the mucilage type determined by its chemical composition. Depending on the mucilage chemical composition, the measured adhesion strength was higher for flax mucilage (dominated by pectins) (91 mN) than for plantain (32 mN) (pectins and some cellulose). We suppose that cellulose fibrils are involved rather in the mucilage stability than in the adhesion process itself [26–27,29]. However, more detailed studies including mucilages with higher content of cellulose (*Ocimum basilicum*, *Salvia hispanica*, and *Lepidium sativum*) gave us further insight into seed mucilage adhesion mechanisms (see below).

### Adhesion of the hydrated mucilage

Biological functions of hydrated mucilage have been described for diverse diaspores. Adhesion under wet conditions appears just after hydration. The mucilage envelope is formed within a few minutes and causes adherence to soil particles. The mass of the diaspore is increasing because of the accumulated water and attached soil particles [123–125]. The total mass increase after the contact of mucilaginous seed to dry soil can increase from 24 times as in *Alyssum minus* [123] to 68 times as in *Lepidium perfoliatum* [123], or even up to 75 times in *Capsella bursa-pastoris* [126]. This mass increase, as well as the adherence of the mucilage to the substrate, may prevent the diaspores from being removed during flooding, water erosion, and/or surface run-off [30,127–128]. Pan et al. [36] tested the effect of erosive surface flow on seeds attached by wet mucilage to the substrate; they summarised that wet (as well as dry) mucilage allowed most species to stay anchored to the substrate. However, the adhesion strength of hydrated mucilage is much lower than that of the dried mucilage envelope [26–27,29]. Yet, the adhesive properties seem to be strong enough for keeping the seed anchored to the soil. A resistance of mucilaginous diaspores against run-off water was also observed in *Helianthemum violaceum* (pectic mucilage) and *Fumana ericifolia* (cellulose mucilage) [30]. The study revealed that this process did not depend on the amount of mucilage produced by the seeds and neither on the chemical composition of the mucilage.

The protective role of the hydrated, viscous mucilage envelope was noted in some studies concerning animal–diaspore interactions. It was observed that mucilage prevents diaspores from predation, because of its viscous character. Seeds with hydrated mucilage are very unwieldy for granivore insects (e.g., ants) collecting seeds [25,129]. Pan et al. [129] observed that workers of harvester ants *Pogonomyrmex subdentatus* were sticking temporarily with their mandibles to mucilaginous seeds or abandoned them. The authors supposed that the mucilage may glue their mouthparts together. Moreover, the sand particles, sticking to the mucilage envelope and camouflaging them, can be an additional factor preventing the seed collection by granivore insects [25,130].

Another example of such sticky traps is described regarding false chinch bugs (*Nysius raphanus*) entrapped by flax seeds covered by the mucilage envelope. These insects stuck on the mucilage when it was dried out [25]. Roberts et al. [131] observed nematodes entrapped by *Capsella bursa-pastoris* seed mucilage. However, in this case, a kind of protocarnivory was supposed, where the dead nematodes could potentially serve as a nutrient source for developing seedlings [131].

The specific chemical composition and spatial structure of the mucilage as well as its adhesive properties, which appear after hydration, are not only important for their biological functions, but also crucial regarding technological applications [17,20]. Adhesion is essential in industrial sectors including medicine, bioengineering, cosmetics, food, and pharmacy [17,20,114,132–134]. Natural polymers present in the seed mucilage are the most attractive source among diverse hydrogels, particularly because of their high biodegradability, non-toxicity, and non-irritability. They also demonstrate attractive bioadhesive properties because of the presence of many carboxyl or hydroxyl groups of polysaccharides, which are the main component of mucilage [17,133–134]. Hydrogels can adhere to diverse inorganic and organic materials under wet conditions [133,135]. Mucilage hydrogels have also the ability to regenerate these properties after rehydration. It was observed that the dried mucilage of *Salvia hispanica* after rehydration fully preserved its adhesive potential and macroscopic structure. This property makes chia mucilage a possible additive ingredient in food production [136].

### Adhesion of the hydrated mucilage dried in contact

Strong permanent adhesion of some plant parts is a well-known phenomenon. English Ivy (*Hedera helix* L.) is a climbing plant able to grow on diverse vertical substrates (trees, walls, and rocks). The plant as many other epiphytes developed attachment roots, which produce a glue-like substance allowing for strong attachment to the substrate [137–139]. This material is composed of pectic polysaccharides and arabinogalactan protein [140]. This glue-like substance is secreted also by other climbing plants (*Parthenocissus quinquefolia* and *Campsis radicans*), which develop special organs, such as tendrils, supporting them in attachment to the substrates [141]. Quantification of the adhesive properties of plant root hairs was done experimentally to give an idea about the adhesive power of the plant. Adventitious roots of *Hedera helix* reached a maximal adhesion (*F*_max_) of 7.07 N, tendrils of *Parthenocissus quinquefolia* (Virginia Creeper) were on the second position with 14.03 N, and the maximal measured value belongs to the tendrils of *Campsis radicans,* namely, 25.18 N [142]. The sea grass (*Posidonia oceanica*) root hairs can also generate strong adhesion under sea water conditions [143–145].

Attachment pads or roots are special organs organised in clusters and supporting the whole plant body climbing on the substrate. In contrast, the seed mucilage envelope is produced by individual diaspores and ensures the diaspore dispersal success or attachment to the ground. Adhesion of hydrated diaspore mucilage reaches values of micronewtons and, as long as water is present in the mucilage, the diaspore can be removed from surfaces (animal fur or feathers). However, even in the fully hydrated state, the mucilage is sticky to ensure the first contact to the surface. Losing the water from mucilage causes stronger adhesion [26–27]; finally, dry mucilage can be strongly cemented to the substrate (glass, soil, or animals) [29]. The results of our studies on adhesive force measurements of dried-in-contact seed mucilage gave us rather unexpected results. The mucilage (of individual seed samples) demonstrated adhesive properties even better than the commercial UHU glue (UHU GmbH & Co. KG, Bühl, Germany). The maximal adhesion (*F*_max_) of the mucilage ranged from 2.03 to 6.22 N [29]. We also tested adhesion of *Plantago ovata* husk (a seed coat covering the seed, built of mucilaginous cells), which reached 37.4 N; the corresponding control samples of UHU glue reached 30.4 N [29].

In our experiments, we observed that the adhesive properties of the mucilage depend on the chemical composition of mucilage, the amount of mucilage produced by the diaspore, the shape and size of the diaspore, and the fractions of polysaccharides and their chemical structure (presence or absence of branched molecules). The spatial structure of the mucilage depends on the presence of side chains attached to the main polysaccharide chain [7,13,23,101]. The mucilage of *Plantago ovata* is composed of highly branched arabinoxylan with side chains that are rich in sites with the affinity to form hydrogen bonds [146]. The diaspores of this taxon also produce an abundant mucilage envelope, which demonstrates strong adhesive properties (5.74 N) [29]. In contrast, *Plantago lanceolata* produces a small mucilaginous envelope, which contains a high amount of unsubstituted xylan backbone making this mucilage less susceptible to bonding with substrates [91] and relatively lowly adhesive (2.03 N) [29].

Cellulose fibrils are present in the mucilage envelope of many plant taxa (e.g., *Ocimum* sp. or *Salvia* sp. *Lepidium* sp.). The highest adhesion of cellulosic mucilage was measured in *Ocimum basilicum* (6.22 N). Basil mucilage comprises thick cellulose fibrils [7]. Cellulose in the form of CNFs is very often used as an additive in the production of, for example, paper and biofilms [41,147–148] because CFNs have many unique properties, such as high mechanical strength (higher than that of steel and alloys, <2 GPa) [149]. Therefore, it was assumed that cellulose fibrils are responsible for the strong mechanical resistance of the mucilage.

Another important physical factor strongly influencing the attachment of mucilaginous seeds to the substrate is the temperature. LoPresti et al. [37] stated that mucilage dried at high temperatures requires, on average, only 33% of the force required to dislodge seeds dried in the refrigerator or at room temperature. It can be supposed that the high temperatures destroy the polysaccharide structure and other important chemical bonds responsible for the interaction between mucilage and substrate. Also, very recent studies involving computer simulations demonstrated the influence of different temperatures on hydrophobic–polar and hydrogen bond interactions within the mucilage envelope [150].

The adhesive strength is expressed as the maximum force per unit area [29,149]; hence, the contact area affects the measured adhesion force. In interspecific comparisons of over fifty species, attachment strength was strongly correlated with the mucilage volume (contact area). Seeds with larger mucilage envelope were attached stronger to the substrate presumably because of the higher contact area [36,129]. It cannot be excluded that many other factors (pH, temperature, humidity, or substrate type) can also influence the adhesive properties of the mucilage envelope.

### The role of mucilage envelope in the seeds dispersal

Fruits and seeds exhibit diverse mechanisms allowing for successful dispersal. Animals contribute in the dispersal of plant diaspores, spreading them over short or long distances [120,151–154]. One of the most effective ways of seed transport by animals is endozoochory. The seeds are ingested, pass through the animal digestive system, and are finally defecated in another place [155–157]. Among diverse animals, birds are an important vector for seeds dispersal particularly on long distances. This strategy can be a strong specialisation of invasive plants, which diaspores are transported to new habitats and quickly start their colonisation [120,152–153,158]. Some factors promote seeds dispersal via endozoochory, that is, a small size and a high number of seeds produced by the plant [159], as well as the presence of mechanisms improving seeds resistance to digestion [154,160]. One of such adaptation, supporting the seeds passage through the digestive system, is the presence of the mucilage envelope. Because of the high slipperiness [26–27], the mucilage envelope should facilitate the passage of the seeds through the bird guts.

As observed in our experiments, from 18900 tested mucilaginous seeds of six plant taxa that were fed to pigeons (*Columa livia domestica*), 841 seeds recovered. From 8100 of non-mucilaginous control seeds of three taxa, only eight recovered. Many seeds were viable and able to germinate. The highest count of seeds was obtained for three *Plantago* taxa (*P. lanceolata*, *P. psyllium*, and *P. ovata*). The result of our studies revealed that the presence of the mucilage envelope supports the endozoochoric transport by birds [28]. The taxa from the genus *Plantago* are distributed on all continents. Many of them exist also as single-island endemics, and this fact is connected with the role of migratory birds as endozoochoric seed dispersers [161]. The ability of mucilage formation helps the diaspores transportation in both dispersal ways, that is, epi- and endozoochoric. *Plantago* seeds were identified in droppings of diverse birds, such as house sparrow (*Passer domesticus*), bullfinch (*Pyrrhula* sp.), greenfinch (*Chloris* sp.), grey partridge (*Perdix perdix*), and racing pigeons (*Columba livia*) [162–166]. This fact also supports the dispersal of *Plantago* seeds via endozoochory.

In our second experiment with the mucilage envelope mechanically removed from the seed surface of three *Plantago* taxa [167] only nine seeds from 8100 seeds fed to pigeons recovered, but none of them germinated. The data from both experiments demonstrate clearly the important role of the mucilage envelope in preventing damage from the digestive system of birds and, thus, in endozoochoric seed dispersal [28,167].

Seeds of non-mucilaginous plants like *Amaranthus retroflexus* and *Chenopodium album* were also found in the droppings of, for example, grey partridge (*Perdix perdix* L.). Approximately 0.3% of the ingested seeds passed the digestive system undamaged, and some of them were able to germinate [158]. In our studies, we tested 2700 *Amaranthus albus* seeds, four of which recovered, and three of them germinated [28]. Such non-mucilaginous seeds are also able to pass the pigeons’ digestive system, but more likely very sporadically. However, even only a few such seeds surviving the way through the birds’ digestive system can have the chance to germinate and to make the first step in the colonisation of a new habitat.

The presence of the mucilage envelope is not the only feature responsible for endozoochoric seeds dispersal. In case of frugivory (fruits dispersal by birds), the dispersed fruits possess some distinct features simplifying their dispersal, among them signalling colour, edible parts, and relatively small size (less than 20 mm) [151]. The fruits can also be equipped with mucilage-like material (hemiparasitic mistletoe *Viscum album*). In mistletoe, the mucilage (viscin) is rather abundant and very sticky, allowing for attachment to tree branches. Diverse birds, such as *Turdus viscivorus* (Mistle Trush), *Bombycilla garrulus* (Bohemian Waxwing), and *Sylvia atricapilla* (Eurasian Blackcap), are known consumers of mistletoe fruits. A number of 16 to 18 fruits can be eaten and pass the digestive system in only 15–20 min. This makes the long distance dispersal of seeds very effective [168]. In their study on long distance seeds dispersal by birds, Viana et al. [169] observed that migratory birds transported seeds over hundreds of kilometres, thus being responsible for the seeds dispersal from the mainland to oceanic islands. Up to 1.2% of the studied birds carried seeds in the guts, and some of the seeds remained viable. If we take into consideration billions of seasonal migratory birds, their effect on seeds transportation over long distances can be rather substantial [169].

## Conclusion

Taking into account that many plants all over the world are able to produce seed/fruit mucilage, we have a natural source of non-toxic, biodegradable hydrogels. The mucilage envelopes of diverse diaspores share many common features, but also demonstrate some differences in their chemical composition and physical character. All the special properties of the mucilaginous envelope (specific composition and 3D architecture, low friction at strong hydration, and high adhesion at low hydration) makes it an important material in diverse industrial applications. Taxa whose mucilage has been studied rather well are *Linum usitatissimum*, *Ocimum basilicum*, *Plantago ovata*, *Salvia hispanica*, and *Lepidium sativum*. However, the number of plant taxa with seeds/fruits able to produce the mucilage envelope and waiting for exploration is huge (mucilaginous seeds/fruits are produced in about 110 different families). Thus, we expect more exciting research on this topic in the near future.

## Data Availability

Data sharing is not applicable as no new data was generated or analyzed in this study.
